# Knowledge of integrated management of childhood illnesses community and family practices (C-IMCI) and association with child undernutrition in Northern Uganda: a cross-sectional study

**DOI:** 10.1186/1471-2458-14-976

**Published:** 2014-09-19

**Authors:** David Mukunya, Samuel Kizito, Tonny Orach, Regina Ndagire, Emily Tumwakire, Godfrey Zari Rukundo, Ezekiel Mupere, Sarah Kiguli

**Affiliations:** Department of Pediatrics and Child Health, College of Health Sciences, Makerere University, Kampala, Uganda; Department of Psychiatry, Mbarara University of Science and Technology, Mbarara, Uganda

**Keywords:** Community and family practices of the integrated management of childhood illnesses, IMCI, Stunting, Wasting, Undernutrition, Sub-Saharan Africa, Gulu

## Abstract

**Background:**

Childhood undernutrition is a major challenge in Uganda with a prevalence of wasting and stunting at 5% and 33%, respectively. Community and family practices of the Integrated Management of Childhood Illnesses (C-IMCI) was introduced in sub-Saharan Africa early after the year 2000. C-IMCI was postulated to address major childhood morbidity and mortality challenges with nutrition as one of the outcomes. The association between knowledge patterns of C-IMCI and undernutrition has not been fully established especially in sub-Saharan Africa. This study was done to address the prevalence of stunting and wasting and the association with the knowledge and practices of C-IMCI among caretakers in Gulu district, Northern Uganda.

**Methods:**

This was a community-based cross-sectional study among 442 caretaker-child pairs. A standardized questionnaire was employed to assess the knowledge and practices of the C-IMCI among caretakers including four practices: breastfeeding, immunization, micronutrient supplementation and complementary feeding. Weight and height of children (6–60 months) were recorded. Wasting and stunting were defined as weight-for-height and height-for-age z-score, respectively, with a cut-off < -2 according to the World Health Organization growth standards. Logistic regression analysis reporting Odds Ratios (OR) with 95% confidence intervals (CI) was used to explore associations using SAS statistical software.

**Results:**

The percentage of caretakers who had adequate knowledge on C-IMCI (basic knowledge within each pillar) was 13%. The prevalence of wasting and stunting were 8% and 21%, respectively. Caretakers’ lack of knowledge of C-IMCI was associated with both wasting (OR 24.5, 95% CI 4.2-143.3) and stunting (OR 4.0, 95% CI 1.3-12.4). Rural residence was also associated with both wasting (OR = 3.1, 95% CI 1.5-6.5) and stunting (OR = 1.7, 95% CI 1.0-2.7). Children younger than 25 months were more likely to be wasted (OR = 3.3, 95% CI 1.7-10.0).

**Conclusion:**

We found a low level of overall knowledge of the C-IMCI of 13.3% (n = 59). There is also a high prevalence of childhood undernutrition in Northern Uganda. Caretakers’ limited knowledge of the C-IMCI and rural residence was associated with both wasting and stunting. Interventions to increase the knowledge of the C-IMCI practices among caretakers need reinforcement.

## Background

Childhood undernutrition is a major health challenge in sub-Saharan Africa with stunting estimated at 40% for boys and 36% for girls [1]. In Uganda, the national level of stunting and wasting among children below five years is 33% and 5%, respectively [2]. In order to reduce childhood mortality and the burden of disease in low and middle income-countries (LMIC), the World Health Organization (WHO) in conjunction with the United Nations’ Children’s Fund (UNICEF) came up with a robust strategy: the Integrated Management of Childhood Illness (IMCI) [3, 4]. IMCI has three components: improving case-management skills of health workers, improving health systems support, and improving family and community practices, also called the community and family practices of IMCI (C-IMCI) [5]. C-IMCI requires the least resources to implement in the IMCI package and has the potential to substantially improve morbidity and mortality among children.

Wasting and stunting were highlighted as the measurable outputs for C-IMCI by the C-IMCI working group [5]. A very strong relationship between poor nutritional indices and morbidity and mortality has been established, causing 2.2million deaths and a fifth of all disability adjusted life years lost worldwide in children less than five years [6]. Stunting has been shown to be the best predictor for nutritional status of children less than five years [7, 8]. On the other hand, wasting is acute undernutrition, influenced by inadequate nutrition and infectious diseases highly prevalent in sub-Saharan Africa [9]. Maternal education may also affect the nutritional status of children [10–15].

Knowledge and practice of C-IMCI among caretakers has been shown to influence children’s nutritional status in studies done in Pakistan and in Bangladesh [16–18], however, there is paucity of related information in sub-Saharan Africa. Gulu district, located in Northern Uganda, is one of the poorest regions in the country which needs sustainable and comprehensive interventions. C-IMCI was pioneered in the district and there is a need to assess its impact. This study was done to determine the level of knowledge and practices of C-IMCI among caretakers and its association with undernutrition in children between 6 and 60 months in Gulu district.

## Methods

### Study setting

The study was conducted in Gulu district approximately 340 km from Kampala city. Gulu has a population of about 385000. Sub-counties near the urban areas have the largest population compared to the rural sub-counties. Gulu district is still recovering from civil strife and people have not fully resettled in the rural areas. The study was conducted in two sub counties; one in the municipality (Pece), with a population of 47000 and one in a rural setting (Bobi), with a population of 22000.

### Study design

The study set out to find the association between knowledge patterns of C-IMCI and undernutrition. The study was a community based cross-sectional study among 442 caretaker-child pairs. The sample size was calculated using the Kish-Leslie formula for single proportions, assuming a knowledge prevalence of 15% [16] among the caretakers, with a design effect of two which was slightly higher than what was used in a similar study [16].

### Sampling

We selected the two sub-counties purposefully. Pece offered an urban setting, while Bobi provided a rural setting. The house with the nearest door to the Local Council one (LC I) Chairperson (village leader) was taken as the index house. Subsequently the house with the nearest door to the previous one was selected provided there was a child between 6 and 60 months and a caretaker present who consented to participate. We aimed at selecting all children within each village, but not everybody was present. Children with a diagnosis of a chronic condition for example sickle cell disease were excluded from the study.

### Data collection

Data was collected in August 2012 by the investigators together with research assistants after one piloting of the study tools. Members of the Village Health Team (VHT) in the respective areas acted as guides to identify the households. We used a modified IMCI-survey questionnaire, a document produced by the IMCI working group [19] to evaluate the effectiveness of the IMCI. The interviews focused on obtaining knowledge and practices of four of the C-IMCI practices (immunization, exclusive breastfeeding, complementary feeding and micronutrient supplementation). Questions regarding knowledge of C-IMCI were open ended and data collectors wrote down all the responses verbatim.

### Anthropometry

We measured weight and height according to a standard WHO protocol [7]. Weight measurements were taken using a digital Seca scale sensitive to ± 0.1 kg with thin or no clothes and no shoes. Children that could stand alone stood, while children who needed to be carried were weighed using the “tare” function. Two readings would be taken and the average recorded. Measurements of length or height were done for children who were shorter or equal to 87 cm (or less than 2 years) in the recumbent position and standing for the rest. The measurements would be read to the nearest 0.1 cm using the Short Infant/Child Height Measuring Board (Short Productions, Woonsocket, RI). Wasting is defined as z-score < -2 weight-for-length/height z-scores and stunting as < -2 length/height-for-age z-score [7]. Using WHO 2006 growth charts the data collectors would record undernutrition of children between 6 and 60 months, which was double-checked. Thus categorized data was used for analysis.

### C-IMCI

We defined our study variables as follows:

Knowledge of Practices: Three key questions were asked to ascertain knowledge of each of the practices being studied. Regarding breastfeeding knowledge, caretakers were asked about recommended period of breastfeeding, advantages of exclusive breastfeeding, and number of times they were advised to breastfeed in a day. As regards complementary feeding knowledge, caretakers were asked the appropriate age of introducing supplementary foods, reasons for complementary feeding and advantages of leafy vegetables to a growing child. With micronutrient knowledge, caretakers were asked the role of vitamin A to a child, period taken before a booster dose of vitamin A is given and the role of iodine to a child. Regarding immunization knowledge, caretakers were asked to give examples of diseases children can suffer from if not immunized, when the first vaccine is supposed to be given, and when the measles vaccine is supposed to be given. Each correctly answered question earned 1 point; this was determined by at least two investigators at the point of data entry with references to predetermined correct responses developed from latest WHO recommendations. A score of 2 or more out of the 3 maximum points was regarded as adequate knowledge of each of the practices. Knowledge of C-IMCI: Adequate knowledge of a minimum of 3 out of the 4 practices studied was regarded as adequate knowledge of C-IMCI. Thus, minimum 50% of the questions were defined as adequate knowledge by our team.

### Data analysis

Data was double entered using Epidata 3.5.1 (http://www.epidata.dk) and analysed using SAS9.2 (Cary software, North Carolina SAS Institute Inc.2004). Descriptive statistics such as frequency, mean, 95% confidence interval (CI), median, and inter quartile range were used to describe data. To determine factors associated with nutritional status, logistic regression was done at both bi-variable and multivariable level. Factors that had a p-value <0.2 and those known from literature review to be associated with nutritional status were included in the multivariable model [16]. The variables included in multivariable analysis were assessed for co-linearity by checking for variance inflation factors (vifs) after fitting the multivariable model. None of the vifs were greater than 10 and the average vif was close to one for both the models using wasting and stunting as the outcomes. We therefore assume that co-linearity is minor.

### Ethical considerations

The research was approved by the Makerere University College of Health Sciences Ethics committee. We obtained written informed consent from the study participants.

## Results

Of the 442 enrolled study participants, 302 (68.3%) were from the urban setting (Pece) and 140 (31.7%) from the rural setting (Bobi). Only 12 (2.7%) of the caretakers were male. Demographic characteristics are shown in Tables 1 and 2.

**Table 1:** **Demographic characteristics of caretakers and children categorical data reported**

Characteristic	Number	Percentage
**Study site**		
Urban	302	68.3
Rural	140	31.7
**Caretakers’ Gender**		
Male	12	2.7
Female	430	97.3
**Education level of caretaker**		
None	55	12.4
Primary	210	47.5
Secondary	120	27.1
Tertiary	57	12.9
**Income of caretaker in Ugandan Shillings** per month*		
<50,000	293	66.3
50,000-200,000	8	1.8
200,000-500,000	40	9.0
>500,000	101	22.9
Head of household income per month*		
<50,000	156	35.3
50,000-200,000	193	43.7
200,000-500000	77	17.4
>500,000	15	3.4
**Head of household sex**		
Male	301	68.1
Female	141	31.9
**Garden Ownership**		
Yes	278	62.9
No	164	37.1
**Full term pregnancy**		
Yes	417	94.3
No	25	5.7
**Sex of child**		
Female	228	51.6
Male	214	48.4
**Twin**		
Yes	17	3.8
No	425	96.2
**Birth weight < 2.5kgs**		
Yes	34	7.7
No	408	92.3

*1 USD = 2500 UgShs 1 Euro = 3000 UgShs

**Table 2:** **Demographic characteristics of caretakers and children longitudinal data reported**

Characteristic	Median	Interquartile range
**Caretakers age, years**	26	22-30
**Head of household age, years**	30	26-36.25
**Number of children in a home**	3	2-4
**Age of child, months**	24	13-36

### Nutritional status of children between 6 and 60 months

The overall prevalence of wasting and stunting among the study children was 8.1% (95% CI: 5.6-10.6) and 21.0% (95% CI: 17.2-24.8), respectively. The prevalence of both wasting and stunting were higher in the rural areas than the urban areas and this finding was statistically significant for wasting (Table 3).

**Table 3:** **Undernutrition frequencies of children between 6 and 60 months in Gulu district by residence and age**

	Wasting	
**Residence**	Yes n (%)	No n (%)
Urban	15(5.00)	287(95.00)
Rural	21(15.00)	119(85.00)
**Age of child**		
<25 months	28(14.00)	217(86.00)
>25 months	8(4.00)	189(96.00)
	**Stunting**	
**Residence**	Yes n (%)	No n (%)
Urban	56(18.50)	246(81.50)
Rural	37(26.40)	103(73.40)
**Age of child**		
<25 Months	52(21.00)	193(79.00)
>25 Months	41(21.00)	156(79.00)

### Knowledge of the C-IMCI

Concerning knowledge of the C-IMCI among the caretakers, 3.8% (n = 17) had adequate knowledge on micro-nutrient supplementation, but within this group only 2.0% (n = 9) had adequate knowledge on Vitamin A supplementation. Regarding feeding, 59% (n = 261) had adequate knowledge on breastfeeding and 17% (n = 75) had adequate knowledge on complementary feeding. Knowledge was better regarding immunization where 85.3% (n = 377) had adequate knowledge. Overall 13.3% (n = 59) had adequate knowledge on the C-IMCI.

Regarding other C-IMCI practices many recommended practices were adhered to by the majority (Table 4), except for boiling drinking water which was only done by 82 (18.6%). The other practices were more common: 207 (67%) had ever de-wormed their children; 414 (93.7%) had ever given their children vitamin A supplements at least once; and 352 (80%) were using insecticide treated mosquito nets.

**Table 4:** **Knowledge and Practices of C-IMCI among caretakers of children under 5 in Gulu district, Uganda**

	Characteristic	Number (n = 442)	Percentage
	Adequate Knowledge		
1	Breastfeeding	261	59
2	Micronutrient	17	3.8
3	Immunization	377	85.3
4	Vitamin A	9	2
5	Complementary feeding	75	17
6	C-IMCI	48	10.9
	Practices		
7	Boiling Drinking Water	82	18.6
8	Deworming	297	67.2
9	Vitamin A supplements	414	93.7
10	ITN usage	352	79.6
11	Iodinated Salt usage	435	98.4
12	Mixed Feeding	84	19

### Factors associated with wasting and stunting

Caretakers’ lack of knowledge on breastfeeding, living in a rural residence, children’s age greater than 25 months, and having a female head of a household were associated with wasting among children less than five years on bi-variable analysis (Tables 5 and 6). On multivariable analysis (Table 7), lack of knowledge of the C-IMCI was significantly associated with wasting (OR = 24.5, 95% CI 4.2-143.3) and stunting (OR = 4.0, 95% CI 1.3 - 12.4). Rural residence was also associated with both wasting (OR = 3.1, 95% CI 1.5-6.5) and stunting (OR = 1.7, 95% CI 1.0-2.7). Children whose mothers had sufficient knowledge on complementary feeding were less likely to be wasted and (OR = 0.0, 95% CI 0–0.2) respectively. Children younger than 25 months where more likely to be wasted (OR = 3.3, 95% CI.1.7-10.0).

**Table 5:** **Wasting and associated factors, bi-variate regression analysis reporting odds ratios, OR, and 95% confidence intervals, CI**

Variable	OR	95% CI
Have Breastfeeding knowledge	2.2	1.0-4.8
Have adequate Knowledge of C-IMCI	1.3	0.5-3.4
Have micronutrient knowledge	2.6	0.7-9.3
Have immunization knowledge	3.1	0.7-13.3
Have vitamin A knowledge	3.4	0.7-16.8
Rural residence	3.3	1.7-6.8
Age category <25 months	3.3	1.4-10.0
Male Child	1.5	0.8-3.1
Male Head of Household	0.3	0.1-0.8
Head of Household Income	0.7	0.3-1.4
Income of caretaker > 1 dollar a day	0.6	0.3-1.4
Mosquito net	2.2	0.7-6.3
Boil water	1.9	0.7-5.6

**Table 6:** **Stunting and associated factors, bi-variate regression analysis reporting odds ratios, OR, and 95% confidence intervals**

Variable	Odds ratio	95% C-I
Have Knowledge of C-IMCI	0.5	0.2-1.1
Have Breastfeeding Knowledge	0.8	0.5-1.3
Have Complementary feeding knowledge	0.8	0.5-1.6
Have Micronutrient knowledge	0.8	0.2-2.8
Have Vitamin A Knowledge	0.5	0.1-3.8
Have Immunization knowledge	1.0	0.5-1.8
Rural residence	1.6	1.0-2.5
Child Age category <25 months	1.0	0.6-1.6
Male Child’s sex	1.1	0.7-1.8
Male Head of Household Income	0.7	0.4-1.1
Male Head of household sex	1.0	0.6-1.7
Income of caretaker <1dollar a day	0.8	0.5-1.3
Care taker education < primary	0.7	0.5-1.2
Sleep under mosquito net	1.0	0.6-1.8
Male Caretaker’s sex	1.3	0.3-6.2

**Table 7:** **Wasting and stunting and associated factors, multivariable regression analysis reporting odds ratios, OR, and 95% confidence intervals**

	Wasting	
Variable	Odds ratio	95% C-I
No Knowledge C-IMCI	24.5	4.2-143.3
Have Complementary Knowledge	0.0	0.0-0.2
Rural Residence	3.1	1.5-6.5
Age category <25 months	3.3	1.7-10.0
Male Child’s sex	1.6	0.8-3.3
Male Head of Household sex	0.4	0.1-1.1
	**Stunting**	
**Variable**	**Odds ratio**	**95% C-I**
No Knowledge of C-IMCI	4.0	1.3-12.4
Have Complementary knowledge	2.1	0.9-5.2
Rural residence	1.7	1.0-2.7
Age category < 25 months	1.0	0.6-1.4
Male Child’s sex	1.1	0.7-1.8
Male Head of household sex	1.0	0.6-1.7

## Discussion

The objective of this study was to determine the caretakers’ knowledge of the C-IMCI and its association with undernutrition of children between 6 and 60 months in Gulu, Uganda.

We found a low level of overall knowledge of the C-IMCI of 13.3% (n = 59). The low level of caretakers’ knowledge on C-IMCI in this area could be due to the low level of education in the northern region with attendance at 51% in primary schools, while the national level is at 81% [2]. This low level of knowledge could also be attributed to the fact that our study was conducted in a post war area hence and many of the people trained in C-IMCI may have relocated to other areas. The low level of C-IMCI could also be due to the knowledge scale we used, which might have been very strict. We recommend public health interventions to reduce the knowledge gap.

We found a high prevalence of wasting and stunting in children in our study. The prevalence of stunting of 26.1% in the rural area is in agreement with the most recent Uganda demographic health survey (UDHS2011) from Northern Uganda in which stunting was reported as 24.7% [2]. However the wasting result of 8.0% is higher than reported in UDHS 2011 of 3.4%. Stunting being a measure of long term nutrition [7] might indicate that our study has been representative for this region. However, our level of wasting was much higher than the level reported by the UDHS. This could be due to a complex of environmental, health, and socio-economic issues which affect wasting. Unfortunately, due to resource constraints, our study did not objectively assess for the presence of acute infections among the study participants, a factor that has been associated with poor weight-for-height z-scores [20, 21].

Stunting and wasting were mainly associated with rural residence and low caretakers’ knowledge of the C-IMCI. This is in agreement with previous studies [16–18, 22] in Pakistan, Bangladesh and Egypt. Knowledge of the C-IMCI could have resulted in a wide range of behavioral change which impacted positively on the nutritional status of the children under care. The association may also be explained by the high formal education more likely found in people with knowledge of the C-IMCI; a finding which we did not appreciate in our study. Formal education in turn has been shown to be associated with better nutritional status among children [10–13, 23]. However, in our study the association between education and better nutritional status was not statistically significant. Socio-economic status was not established in our study but a number of studies have shown that it is associated with nutritional status [14, 21, 23–25]. The relationship between rural residence and poor nutritional status which has also been found by previous authors [26] could be because most people in rural areas have lower socioeconomic status. It could also be because rural areas tend to have low standards of living which would explain the poor nutritional indices. Children less than 25 months were more likely to be wasted; this is probably due to the possibility of a higher infectious disease burden in this age group, in this particular region. We also found very low knowledge on vitamin A and Zinc supplementation followed by poor practices of vitamin A supplementation. This could be because the role of micronutrients has only recently been emphasized in this region. The low number of people who boil drinking water in this region is probably due to the fact that most people get their water from boreholes which are wrongly believed to have safe water [27].

Our study was a community study in a sub-Saharan setting with strengths and weaknesses. Of the strengths, it focused on one pillar of IMCI which is C-IMCI that is less studied. We also see it as a strength that we used open ended questions to address knowledge and believe the answers given by the caretakers were valid as they seemed secure in the discussion. However, post-data collection classification errors cannot be ruled out. Due to the fact that this was a cross-sectional study, causation cannot be assessed. As we studied only four components of the C-IMCI, we could not rule out other causes of poor nutritional indices like recent infections. We believe that our sample was representative for the district, though we cannot rule out that the use of VHT and lack of censuses for random selection of participants can have contributed to selection bias.

## Conclusion

We found a very low level of knowledge of C-IMCI among caretakers of children between 6 and 60 months in Gulu, Uganda. Limited knowledge of C-IMCI was associated with both wasting and stunting. Interventions to increase the caretakers’ knowledge of C-IMCI need reinforcement.
